# Supplementary material for: Clinical characteristics and primary management of patients diagnosed with prostate cancer between 2015 and 2019 at the Uganda Cancer Institute
Source: PLoS One. 2020 Oct 30;15(10):e0236458. doi: 10.1371/journal.pone.0236458 (PMC7598473; doi:10.1371/journal.pone.0236458)
Supplement: Attachment — Submitted filename: Response to Reviewers comments_Paul Katongole.docx [file pone.0236458.s002.docx]

**The Academic Editor**

**PLOS ONE**

**Lucia R Languino, Ph.D.**

**12^th^ October 2020**

**Dear,**

**RE; RESPONSE TO REVIEWERS COMMENTS**

I am grateful for the opportunity given to review my submitted manuscript entitled; Clinical characteristics and primary management of patients diagnosed with prostate cancer between 2015 and 2019 at the Uganda Cancer Institute.

After carefully reading and internalizing the comments from the reviewers, below are the responses;

**Reviewer 1#**

1. In line 84-85, we used the ESMO classification of localized prostate cancer. Under this classification locally advanced prostate cancer is under category of high risk T3-T4. We have also added this to the text in line 86.
2. In line 105 -107 we have included a statement; Poor documentation could not allow the classification into metastatic castration naïve prostate cancer (mCNPC), castration sensitive prostate cancer (CSPC) or metastatic castration resistant prostate cancer (mCRPC).
3. In line 119 to 121, the statement has been corrected as below; the most commonly used chemotherapeutic agents were Cabazitaxel and docetaxel, while bicalutamide was the main homonotherapeutic agent used.
4. In line 142, the sentence has been changed and we removed the phrase “In this study” in order to reduce monotony.
5. In line 145 to 147, the statement has been rephrased to remove immunotherapy as below; this is compounded by the limited human resource and the limited number of technologically advanced diagnostic and treatment modalities like modern radiotherapy machines and next generation sequencing.
6. In line 149 to 151, we have changed the word chemotherapy to Androgen deprivation therapy.
7. The conclusion has been rephrased as follows; In this study majority (99%) of the men were diagnosed with advanced prostate cancer. There is therefore need for improved access to prostate cancer screening, diagnosis and treatment in the different regions, through the setup of regional cancer treatment centers in order to improve access to care. Community engagement and community based prostate cancer screening and diagnosis campaigns could be a possible solution to early diagnosis and linkage to care.

**Reviewer 2#**

1. Percentages have been added to the columns in table 1 and in table 3, the numbers were errors due to editing and have been removed.

**Funding information**

In regards to funding for this manuscript this should the updated statement;

PK receives funding from the African Development bank under Uganda Cancer institute support PK's doctoral work and also support from a training grant ,D43TW010132 supported by Office of the Director, National Institutes of Health (OD), National Institute of Dental & Craniofacial Research (NIDCR), National Institute of Neurological Disorders and Stroke (NINDS), National Heart, Lung, And Blood Institute (NHLBI), Fogarty International Center (FIC), National Institute on Minority Health and Health Disparities (NIMHD). Funded in part (SJR) by the Division of Intramural Research, National Institute of Allergy and Infectious Diseases (NIAID). Its contents are solely the authors' responsibility and do not necessarily represent the supporting offices' official views.

Sincerely

Dr. Paul Katongole

[paul.katongole@gmail.com](mailto:paul.katongole@gmail.com)
