# Supplementary material for: Clinical characteristics and primary management of patients diagnosed with prostate cancer between 2015 and 2019 at the Uganda Cancer Institute
Source: PLoS One. 2020 Oct 30;15(10):e0236458. doi: 10.1371/journal.pone.0236458 (PMC7598473; doi:10.1371/journal.pone.0236458)
Supplement: Peer review history

# Peer review: Decision Letter 0

**DOI:** 10.1371/journal.pone.0236458.r001

**Authors:** Lucia R. Languino

7 Oct 2020

PONE-D-20-20673

Clinical characteristics and primary management of patients diagnosed with prostate cancer between 2015 and 2019 at the Uganda Cancer Institute

PLOS ONE

Dear Dr. Katangole,

Thank you for submitting your manuscript to PLOS ONE. After careful consideration, we feel that it has merit but does not fully meet PLOS ONE’s publication criteria as it currently stands. Therefore, we invite you to submit a revised version of the manuscript that addresses the points raised during the review process.

Please submit your revised manuscript by Nov 21 2020 11:59PM. If you will need more time than this to complete your revisions, please reply to this message or contact the journal office at plosone@plos.org. When you're ready to submit your revision, log on to https://www.editorialmanager.com/pone/ and select the 'Submissions Needing Revision' folder to locate your manuscript file.

Please include the following items when submitting your revised manuscript:

- A rebuttal letter that responds to each point raised by the academic editor and reviewer(s). You should upload this letter as a separate file labeled 'Response to Reviewers'.
- A marked-up copy of your manuscript that highlights changes made to the original version. You should upload this as a separate file labeled 'Revised Manuscript with Track Changes'.
- An unmarked version of your revised paper without tracked changes. You should upload this as a separate file labeled 'Manuscript'.

If you would like to make changes to your financial disclosure, please include your updated statement in your cover letter. Guidelines for resubmitting your figure files are available below the reviewer comments at the end of this letter.

If applicable, we recommend that you deposit your laboratory protocols in protocols.io to enhance the reproducibility of your results. Protocols.io assigns your protocol its own identifier (DOI) so that it can be cited independently in the future. For instructions see: http://journals.plos.org/plosone/s/submission-guidelines#loc-laboratory-protocols

We look forward to receiving your revised manuscript.

Kind regards,

Lucia R. Languino, Ph.D.

Academic Editor

PLOS ONE

Journal Requirements:

When submitting your revision, we need you to address these additional requirements.

1. Please ensure that your manuscript meets PLOS ONE's style requirements, including those for file naming. The PLOS ONE style templates can be found at

https://journals.plos.org/plosone/s/file?id=wjVg/PLOSOne_formatting_sample_main_body.pdf and

https://journals.plos.org/plosone/s/file?id=ba62/PLOSOne_formatting_sample_title_authors_affiliations.pdf

2.Thank you for stating the following in the Funding Section of your manuscript:

[Funds from the African Development bank under Uganda Cancer institute support PK's doctoral

191 work. PK also is receiving support from a training grant and by Grant Number D43TW010132

192 supported by Office of the Director, National Institutes of Health (OD), National Institute of

193 Dental & Craniofacial Research (NIDCR), National Institute of Neurological Disorders and

194 Stroke (NINDS), National Heart, Lung, And Blood Institute (NHLBI), Fogarty International

195 Center (FIC), National Institute on Minority Health and Health Disparities (NIMHD). It was

196 funded in part (SJR) by the Division of Intramural Research, National Institute of Allergy and

197 Infectious Diseases (NIAID). Its contents are solely the authors' responsibility and do not

198 necessarily represent the supporting offices' official views.]

We note that you have provided funding information that is not currently declared in your Funding Statement. However, funding information should not appear in the Acknowledgments section or other areas of your manuscript. We will only publish funding information present in the Funding Statement section of the online submission form.

Please remove any funding-related text from the manuscript and let us know how you would like to update your Funding Statement. Currently, your Funding Statement reads as follows:

 [There is no particular funding for this work]

Please include your amended statements within your cover letter; we will change the online submission form on your behalf.

3. We note that you have stated that you will provide repository information for your data at acceptance. Should your manuscript be accepted for publication, we will hold it until you provide the relevant accession numbers or DOIs necessary to access your data. If you wish to make changes to your Data Availability statement, please describe these changes in your cover letter and we will update your Data Availability statement to reflect the information you provide.

4. Your ethics statement should only appear in the Methods section of your manuscript. If your ethics statement is written in any section besides the Methods, please delete it from any other section.

5. Please include captions for your Supporting Information files at the end of your manuscript, and update any in-text citations to match accordingly. Please see our Supporting Information guidelines for more information: http://journals.plos.org/plosone/s/supporting-information.

[Note: HTML markup is below. Please do not edit.]

Reviewers' comments:

Reviewer's Responses to Questions

**Comments to the Author**

1. Is the manuscript technically sound, and do the data support the conclusions?

The manuscript must describe a technically sound piece of scientific research with data that supports the conclusions. Experiments must have been conducted rigorously, with appropriate controls, replication, and sample sizes. The conclusions must be drawn appropriately based on the data presented. 

Reviewer #1: Partly

Reviewer #2: Yes

**********

2. Has the statistical analysis been performed appropriately and rigorously? 

Reviewer #1: Yes

Reviewer #2: I Don't Know

**********

3. Have the authors made all data underlying the findings in their manuscript fully available?

The PLOS Data policy requires authors to make all data underlying the findings described in their manuscript fully available without restriction, with rare exception (please refer to the Data Availability Statement in the manuscript PDF file). The data should be provided as part of the manuscript or its supporting information, or deposited to a public repository. For example, in addition to summary statistics, the data points behind means, medians and variance measures should be available. If there are restrictions on publicly sharing data—e.g. participant privacy or use of data from a third party—those must be specified.

Reviewer #1: Yes

Reviewer #2: Yes

**********

4. Is the manuscript presented in an intelligible fashion and written in standard English?

PLOS ONE does not copyedit accepted manuscripts, so the language in submitted articles must be clear, correct, and unambiguous. Any typographical or grammatical errors should be corrected at revision, so please note any specific errors here.

Reviewer #1: Yes

Reviewer #2: Yes

**********

5. Review Comments to the Author

Please use the space provided to explain your answers to the questions above. You may also include additional comments for the author, including concerns about dual publication, research ethics, or publication ethics. (Please upload your review as an attachment if it exceeds 20,000 characters)

Reviewer #1: 1. Please clarify in the methodology (Line 84-85) that:

The risk stratification is designed mostly for localized prostate cancer; classified as either Low risk: cT1-2a, PSA<10, GS-6; intermediate-risk: cT2b-c, PSA 10–20, GS 7; High-risk: T3-T4, PSA>20. Locally advanced cancer is also designated as high risk.

2. The study results showed that 99.12% of prostate cancer patients presented with advanced with advanced disease. It would have been essential to document various cadre of metastatic prostate cancer like Metastatic Castration-Naïve Prostate cancer (mCNPC), Castrate-sensitive prostate cancer (CSPC) or Metastatic Castrate-Resistant Prostate cancer and various treatment options (mCRPC). If not, you can mention that it was possible due to poor documentation.

3. In the Results line 115 and 116, the authors have confused hormonotherapy (bicalutamide) with chemotherapeutic agents (docetaxel and taxanes); please rectify.

4. In line 135 and 138, both sentences subsequently began with ‘in this study’. Please rephrase to reduce monotony.

5. In line 142 to 144, you mentioned the lack of immunotherapy as a probable limitation to prostate cancer care. To date there is insufficient evidence to the suggest the role of immunotherapy in the management of prostate cancer. Please rectify.

6. Regarding line 146 and 149, enzalutamide and bicalutamide are better considered as androgen deprivation therapy or hormonotherapy when managing prostate cancer. Avoid referring to them as chemotherapy.

7. Please rephrase the conclusion to present a take home message or recommendation; it is currently structured like an abstract. Ex. The first sentence of the conclusion may be replaced with a finding or recommendation.

Reviewer #2: In your table 1, it would be helpful if you included percentages as well in the column to the right in addition to the raw numbers. In table 3, in two rows of the column to the right there are extra numbers to the side that is unclear what they refer to.

**********

6. PLOS authors have the option to publish the peer review history of their article (what does this mean?). If published, this will include your full peer review and any attached files.

If you choose “no”, your identity will remain anonymous but your review may still be made public.

**Do you want your identity to be public for this peer review?** For information about this choice, including consent withdrawal, please see our Privacy Policy.

Reviewer #1: No

Reviewer #2: **Yes: **Mark Mann

[NOTE: If reviewer comments were submitted as an attachment file, they will be attached to this email and accessible via the submission site. Please log into your account, locate the manuscript record, and check for the action link "View Attachments". If this link does not appear, there are no attachment files.]

While revising your submission, please upload your figure files to the Preflight Analysis and Conversion Engine (PACE) digital diagnostic tool, https://pacev2.apexcovantage.com/. PACE helps ensure that figures meet PLOS requirements. To use PACE, you must first register as a user. Registration is free. Then, login and navigate to the UPLOAD tab, where you will find detailed instructions on how to use the tool. If you encounter any issues or have any questions when using PACE, please email PLOS at figures@plos.org. Please note that Supporting Information files do not need this step.

# Author comment: Author response to Decision Letter 0

**DOI:** 10.1371/journal.pone.0236458.r002

14 Oct 2020

The responses have been included in the cover letter

Attachment Submitted filename: Response to Reviewers comments_Paul Katongole.docx Click here for additional data file.

# Editor report: Decision Letter 1

**DOI:** 10.1371/journal.pone.0236458.r003

**Authors:** Lucia R. Languino

19 Oct 2020

Clinical characteristics and primary management of patients diagnosed with prostate cancer between 2015 and 2019 at the Uganda Cancer Institute

PONE-D-20-20673R1

Dear Dr. KATONGOLE,

We’re pleased to inform you that your manuscript has been judged scientifically suitable for publication and will be formally accepted for publication once it meets all outstanding technical requirements.

Within one week, you’ll receive an e-mail detailing the required amendments. When these have been addressed, you’ll receive a formal acceptance letter and your manuscript will be scheduled for publication.

An invoice for payment will follow shortly after the formal acceptance. To ensure an efficient process, please log into Editorial Manager at http://www.editorialmanager.com/pone/, click the 'Update My Information' link at the top of the page, and double check that your user information is up-to-date. If you have any billing related questions, please contact our Author Billing department directly at authorbilling@plos.org.

If your institution or institutions have a press office, please notify them about your upcoming paper to help maximize its impact. If they’ll be preparing press materials, please inform our press team as soon as possible -- no later than 48 hours after receiving the formal acceptance. Your manuscript will remain under strict press embargo until 2 pm Eastern Time on the date of publication. For more information, please contact onepress@plos.org.

Kind regards,

Lucia R. Languino, Ph.D.

Academic Editor

PLOS ONE

Additional Editor Comments (optional):

Reviewers' comments:

# Editor report: Acceptance letter

**DOI:** 10.1371/journal.pone.0236458.r004

**Authors:** Lucia R. Languino

22 Oct 2020

PONE-D-20-20673R1 

Clinical characteristics and primary management of patients diagnosed with prostate cancer between 2015 and 2019 at the Uganda Cancer Institute 

Dear Dr. Katongole:

I'm pleased to inform you that your manuscript has been deemed suitable for publication in PLOS ONE. Congratulations! Your manuscript is now with our production department. 

If your institution or institutions have a press office, please let them know about your upcoming paper now to help maximize its impact. If they'll be preparing press materials, please inform our press team within the next 48 hours. Your manuscript will remain under strict press embargo until 2 pm Eastern Time on the date of publication. For more information please contact onepress@plos.org.

If we can help with anything else, please email us at plosone@plos.org. 

Thank you for submitting your work to PLOS ONE and supporting open access. 

Kind regards, 

PLOS ONE Editorial Office Staff

on behalf of

Dr. Lucia R. Languino 

Academic Editor

PLOS ONE
